# Supplementary material for: Evaluation of rat pancreatic islets damage after siRNA microporation
Source: PLoS One. 2026 Jan 23;21(1):e0340066. doi: 10.1371/journal.pone.0340066 (PMC12829790; doi:10.1371/journal.pone.0340066)
Supplement: Attachment — Submitted filename: Response to reviewers.pdf [file pone.0340066.s002.pdf]

INSTITUTE FOR CLINICAL AND EXPERIMENTAL MEDICINE
Department of Diabetes
HEAD: assoc. prof. Peter Girman, Ph.D., M.D.
In charge: assoc. prof. Jan Kriz, Ph.D., M.D., tel./e-mail: +420261364107, jkri@ikem.cz

10/10/2025 in Prague, CZ

To the Editors and reviewers,
PLOS ONE

Subject: The answer to editors of submitted manuscript entitled **"Evaluation of *rat* pancreatic islets damage after siRNA microporation"**

Dear Editor and reviewers,

On behalf of my co-authors, I am authorized to send you a carefully prepared answer and reedited manuscript. First of all, I would like to thank the reviewers for carefully reading the manuscript and providing valuable comments on the text and the clarity of the figures. We believe maximum comprehensibility for readers to be an essential condition for an article to be considered useful. Our responses to reviewer's comments and requests are as follows:

Reviewer #1:

1. Please indicate to the aim of the study in introduction section, remove the last 3 sentences about what you did.
The last paragraph of the Introduction was rephrased as requested (lines 87-95 in tracked changes file).

2. Use the same font for title and text.
The fonts were part of the Word settings, and their purpose was to highlight individual heading levels. I'm not sure if this is a significant error, given that the publisher will modify all fonts for printing anyway to match the journal layout, but we unified the fonts according to the reviewer's request.

3. In method section, provide explanation about the methods which used by detail and write continuously in one paragraph foreach method, not just mention as i. ii, etc.
The method Section: „Evaluation of pancreatic islets after treatment" contains a list of methods used under letters i., ii., etc. for clarity. The individual methods are described in detail in the following text, as requested by the reviewer.

4. Indicate to the cell number which used for transfection.
The text was rephrased as requested (lines 137, 146 in tracked changes file).

5. Indicate to the house keeping gene for q-RT-PCR evaluation.
The text was rephrased as requested (lines 213 in tracked changes file).

6. Indicate to the software you used for statistical analysis.
The text was rephrased as requested (lines 248, 249 in tracked changes file).

7. Indicate to the exact value of p value in results.
The text was rephrased as requested (lines 337-341, 366, 466, 467, 470, 471, 513-514 in tracked changes file).

P-values: If any statistical significance was stronger than $p < 0.001$, the exact number is not written by the software.

8. The conclusion section contain redundancy, rewrite this part.
The text of Conclusion section was rephrased as requested. The redundant information was erased and the suggested contribution to other groups was added (lines 535 - 545).

9. In conclusion section, you should just indicate to the main findings and decision you got of the study. You should not indicate to the references.

The text of Conclusion section was rephrased as requested. The redundant information was erased and the suggested contribution to other groups was added (lines 535 - 545).

10. The lack of comparison between your findings and previous studies has been found in discussion section.

Our feeling is the same, but the problem is, that there are not several studies using microporation for transfection of molecules to pancreatic islet cells. The comparison to basic electroporation or lipofection is not reliable. But we tried to do our best and add some references to the text of **"Results and discussion"** section as follows:

Line 382 the Lefebvre paper (40) is the newest paper of this problematic we have found
Line 384 the reference of Nico DeLeu 2014 was added and the sentence slightly rephrased
Line 397 the reference of Garcia-Sastre 2017 was added
Line 439, 440 the reference of El-Zayat 2019 et al was added
Line 450 the reference of Lee 2020 was added

11. Indicate to the primer sequences in the table.

All primers TaqMan qRT PCR analysis are mentioned in methods section with specific ID in catalogue of ThermoFisher® Scientific company. The sequences were not provided by the supplier as it is a protected know-how of the company. The genomic maps of target mRNA and symbolic location of the primer were provided.

12. Most of the references are out of date, try to use references in recent 10 years.

We added several current references as requested

Reviewer #2 - major comments:

Figure 1

- **Update the titles of columns A and B to indicate the magnification used for image acquisition (e.g., 10×, 20×).**
- **Scale bars: Ensure that scale bars are visible, consistent, and clearly labeled across all panels. The grey scale bar in column A is barely noticeable, and column B appears to lack one. For columns C and D, the small text below the scale bars is unreadable; I recommend removing this text and instead reporting scale bar lengths in the figure caption.**
- **Rename the titles of columns C and D to something more descriptive, such as "Viability (PI/DAPI?)" and "Apoptosis (Caspase3/7)".**
- **Color coding: Specify what each color represents (e.g., green, red, blue) for the stains used. This information should be provided either directly in the figure legend or in the caption for clarity.**
- **Optimize layout: Reduce the excessive spacing between images to improve figure readability.**

The picture was edited according to request of the reviewer. Description of scale bars was moved from the picture to the picture legenda, titles were renamed and colors are explanted in the picture description.

Figure 2

- **Explicitly state on the figure caption what the black dots and vertical lines represent in the plots.**
- **Replot the data using transparent boxplots to show group averages, and overlay individual replicate values as jittered points for clarity.**
- **High-resolution data representation: The manuscript states that quantitative analyses were performed by three independent evaluators across four separate experiments. This rich dataset could be presented more effectively by including on the plots:**
  - **Individual replicates**
  - **Evaluator contributions**
  - **Group averages**
  - **Statistical test results**
- **Guidelines for visualization: I recommend consulting Lin & Landry (2024; https://www.biorxiv.org/content/10.1101/2024.09.20.609464v2) for best practices in boxplot data visualization.**
- **Formatting adjustments: Increase the size of axis titles, tick labels, and asterisks indicating statistical significance to improve readability.**

Vídeňská 1958/9
140 21 Praha – Krč
IČ: 00023001
DIČ: CZ00023001
E-mail: jan.kriz@ikem.cz
Tel.: +420 261 364 107
Fax: +420 261 362 820
www.ikem.cz

The graph and its description were modified by the request of the reviewer 2. The statistician modified it as much as possible to bar plots in order to keep it correct.

Figure Captions (General)
The figure captions are currently difficult to follow and should be restructured for greater clarity and consistency:

- **Group related panels logically: For example, in Fig. 1, combine columns A+B as Fig. 1A, column C as Fig. 1B, and column D asFig. 1C.**
- **Focus on describing the content: Captions should state what is being shown (e.g., variables, treatments, measurements, stats) rather than primarily summarizing results. If desired, key findings can be highlighted in the figure title instead.**
- **Include statistical details: Report relevant information (e.g., tests performed, sample sizes, p-values) within the captions themselves, not only in the Methods section.**
- **I recommend reviewing Jambor et al. (2021; https://journals.plos.org/plosbiology/article?id=10.1371/journal.pbio.3001161) and Liu et al. (2023; https://www.sciencedirect.com/science/article/abs/pii/S0889490622000606) for figure legend description.**

Thank you for this comment. We have standardized the graph labels and focused more consistently on describing the information displayed in the graphs with an indication of the intervention used. The font is significantly larger to make it easier to read. We hope, the reviewer would feel it improved.

Figure 3

- **Apply the same recommendations as for Figure 2:**
- **Use transparent boxplots with jittered replicates.**
- **Increase font sizes for axis titles, tick labels, and statistical annotations.**

The graph and its description were modified by the request of the reviewer. The statistician modified it as much as possible to bar plots in order to keep it correct.

Figures 4–5

- **If possible, increase the font size for all labels and annotations to improve readability.**

Was done as requested.

Figure 6

- **Replot the data using the same recommendations as for Figures 2 and 3:**
- **Transparent boxplots**
- **Jittered replicates- Inclusion of statistical information and additional data**

The graph and its description were modified by the request of the reviewer. The statistician modified it as much as possible to bar plots in order to keep it correct.

Reviewer #2 - minor comments:

- **Line 89: Correct "Ratus norvergicus" → Rattus norvegicus (capitalize R).**

Done

- **Line 274: Rephrase for clarity: "Poly(I:C) is a known activator of inflammation."**

Done

- **Line 533: Update phrasing using "on", "…caused any negative effects on pancreatic islets…".**

The sentence was completely replaced according to request of the reviewer 1.

Vídeňská 1958/9
140 21 Praha – Krč
IČ: 00023001
DIČ: CZ00023001
E-mail: jan.kriz@ikem.cz
Tel.: +420 261 364 107
Fax: +420 261 362 820
www.ikem.cz

- **Line 535: Improve the ending of the phrase by giving the research overall purpose: "…reliably employed in future experimental studies for treating some patients with type 1 diabetes mellitus."**

Done

At the end I would like to state that the founders had no role in study design, data collection and analysis, decision to publish, or preparation of the manuscript.

Sincerely yours,

Jan Kriz

Jan Kříž

Digitálně podepsal Jan Kříž
Datum: 2025.10.09 18:54:54 +02'00'

assoc. prof. Jan Kriz, MD, PhD
Institute for Clinical and Experimental Medicine
Diabetes Center
Videnska 1958/9
140 21 Prague 4, Czech Republic
Phone: +420 261364107
Email: jkri@ikem.cz
